# Effects of resistance exercise training on depressive symptoms among young adults: A randomized controlled trial

**DOI:** 10.1016/j.psychres.2023.115322

**Published:** 2023-06-28

**Authors:** Darragh O’Sullivan, Brett R. Gordon, Mark Lyons, Jacob D. Meyer, Matthew P. Herring

**Affiliations:** a Department of Physical Education and Sport Sciences, University of Limerick, Limerick, Ireland; b Physical Activity for Health Research Centre, Health Research Institute, University of Limerick, Limerick, Ireland; c Public Health Sciences, Penn State College of Medicine, Pennsylvania, United States; d Sport and Human Performance Research Centre, University of Limerick, Limerick, Ireland; e Department of Kinesiology, Iowa State University, Iowa, United States

**Keywords:** Muscle strengthening exercise, Resistance training, Depression, Generalized anxiety disorder, Young adults

## Abstract

Evidence supports the antidepressant effects of resistance exercise training (RET); however, findings among young adults at-risk for elevated depressive symptoms are limited. This randomized controlled trial examined the effects of eight weeks of ecologically-valid, guidelines-based RET, compared to a wait-list control, on depressive symptoms among 55 young adults (26±5y; 36 female) with and without subclinical, or analogue, Generalized Anxiety Disorder (AGAD; Psychiatric Diagnostic Screening Questionnaire GAD subscale ≥6 and Penn State Worry Questionnaire ≥45) and Major Depressive Disorder (AMDD). Following a three-week familiarization period, participants completed one-on-one, twice-weekly RET sessions. The 16-item, self-reported Quick Inventory of Depressive Symptomatology (QIDS) assessed depressive symptoms. RM-ANCOVAs examined between-group differences, and significant interactions were decomposed with simple effects analysis. Hedges’ *d* effect sizes (95%CI) quantified the magnitude of differences in change between groups across time. Stratified analyses were conducted among subsamples with AMDD and AGAD. There were no baseline depressive symptom differences between groups. Attendance was 83%, and compliance was 80%. RET induced statistically significant, clinically-meaningful, large-magnitude reductions in depressive symptoms from baseline to week eight in the total (*d* = 1.01; [95%CI: 0.44–1.57]), AMDD (*d* = 1.71; [95%CI: 0.96–2.46]), and AGAD (*d* = 1.39; [95%CI: 0.55–2.24]) samples. These findings support guidelines-based RET as a promising treatment for mild depression.

## 1. Introduction

Depression has a lifetime prevalence of approximately 15.4% (Lim et al., 2018). It is characterized by symptoms including depressed mood, loss of interest and/or pleasure, poor concentration, and disturbed sleep or appetite (National Institute for Health and Care Excellence 2022). Depression is associated with increased risk of suicide (Bernal et al., 2007), all-cause mortality (Cuijpers and Smit, 2002), cardiovascular disease (Correll et al., 2017), Alzheimer’s disease (Green et al., 2003), social dysfunction (Fombonne et al., 2001), and a major economic burden, costing approximately €118 billion per annum in Europe (Sobocki et al., 2006). Approximately 55% of people with depression receive contact with primary care or specialist services, of which only 32% receive adequate treatment (Andrews et al., 2000). First-line depression treatments include medication and psychotherapy. The effectiveness of antidepressant medications increase with greater depression severity, but these medications have minimal effectiveness for mild-to-moderate depressive symptoms (Cohen’s d = 0.11; number needed to treat (NNT)=16) (Fournier et al., 2010). Negative side-effects of antidepressant medications can also cause poor treatment adherence and low tolerability (Wang et al., 2018). Psychotherapy, while effective (Cuijpers et al., 2020), can also be expensive and inaccessible. Consequently, alternative and augmentative treatments, such as exercise training, are needed for depression.

The antidepressant effects of exercise are well-documented (Herring et al., 2012; Schuch et al., 2016), and are underpinned by several biologically plausible mechanisms. These mechanisms include improved regulation of the monoaminergic system (Dishman et al., 2006) and stress-response (Sheline et al., 2003; Duman, 2004), reductions in inflammatory markers (Olson et al., 2007), and increased IGF-1 (Cassilhas et al., 2010). The overwhelming majority of interventions that have examined the effects of exercise on depressive symptoms have used aerobic exercise training (AET). The promising effects of resistance exercise training (RET) on depressive symptoms remain understudied (Garber et al., 2011; Cooney et al., 2013; Netz, 2017). Recent meta-analytic evidence showed that RET significantly reduces depressive symptoms among otherwise healthy (mean effect size delta (Δ)=0.81) and mentally-ill adults (Δ=1.00) (Gordon et al., 2018). Only three trials focused on young adults (*d* = 0.52 to 1.48) (Doyne et al., 1987; Herring et al., 2011; Vizza et al., 2016), all of which included participants with baseline depressive symptoms indicative of at least mild depression. One trial examined depressive symptom changes among participants with clinically-meaningful anxiety (*d* = 0.52) (Herring et al., 2011). This lack of research among young adults, and those with clinically-meaningful anxiety, are intertwined limitations of the available evidence.

Depressive disorders typically develop later in adulthood. However, the median age of onset has progressively declined (Kessler et al., 2003), and approximately 36.9% of cases first develop before 25 years of age (Solmi et al., 2022). Additionally, GAD typically develops in young adulthood (Baxter et al., 2013, 2014), and is highly comorbid with Major Depressive Disorder (MDD) (Lieb et al., 2005), suggesting that young adults with clinically-meaningful anxiety may be at-risk for elevated or clinically-meaningful depressive symptoms. The frequent comorbidity of depression and anxiety, and lack of evidence of the effects of RET, warrants a greater focus on these populations.

Heterogeneity of the design and protocols of RET interventions is another limitation that potentially limits the widespread prescription of RET as a therapy for depression. The World Health Organization (WHO) and American College of Sports Medicine (ACSM) muscle-strengthening guidelines provide a widely implementable, ecologically-valid RET option. The WHO recommends engaging in muscle-strengthening activities involving major muscle groups on two or more days per week (WHO, 2011). More detailed guidelines from ACSM recommend progressive strength training on two to three days per week, with 2–3 sets of 8–12 repetitions for muscle strength benefits among individuals with limited or no RET experience (ACSM, 2019). RET designed in accordance with ACSM guidelines has demonstrated anxiolytic effects among young adults with (*d* = 0.71) (Gordon et al., 2021) and without (*d* = 0.85) (Gordon et al., 2020b) clinically-meaningful anxiety.

Therefore, this secondary analysis of existing randomized controlled trial (RCT) data extends past reports by quantifying the effects of ecologically-valid, guidelines-based RET on depressive symptoms among young adults with and without subclinical, or analogue, Generalized Anxiety Disorder (AGAD) and Major Depressive Disorder (AMDD).

## 2. Methods

This pilot efficacy trial adhered to the Consolidated Standards of Reporting Trials (CONSORT) Checklist (Schulz et al., 2010).

### 2.1. Trial design

Secondary analyses of existing data from two parallel, eight-week RCTs (ClinicalTrials.gov Identifier: NCT04116944) were completed; the full methods (Gordon et al., 2020a) and primary outcomes (Gordon et al., 2020b, 2021) are published. The research protocol was approved by the University’s Research Ethics Committee (EHSREC No: 2017_03_18_EHS); all participants provided written informed consent prior to participation which was not compensated. This trial had rolling recruitment; data collection commenced in January 2018, and concluded in June 2019.

### 2.2. Participants

**Fig. 1.**
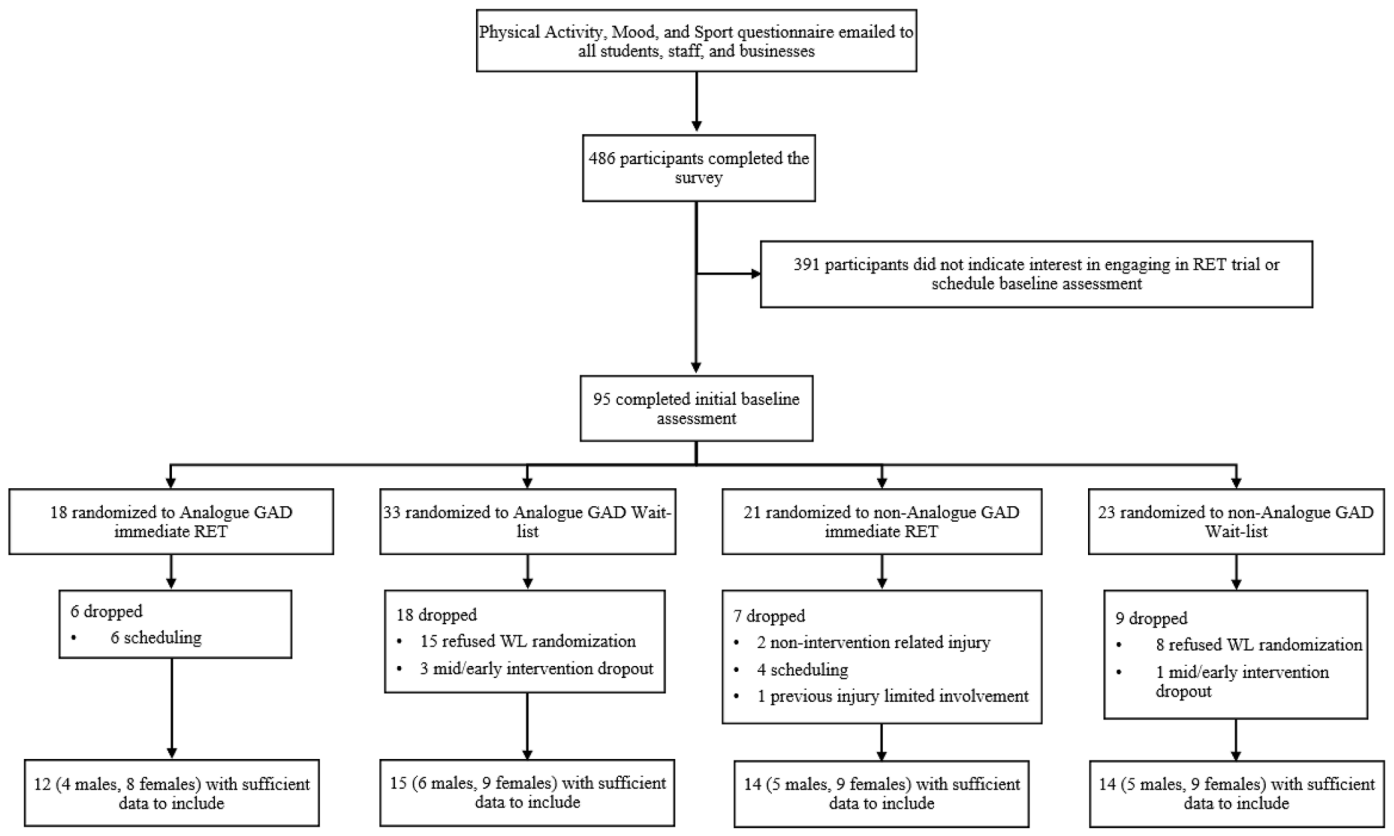
flowchart of participant recruitment.

Participants were recruited via posters, emails, and word of mouth, and initially completed an electronic battery of questionnaires to establish eligibility; Fig. 1 presents a flowchart of participant recruitment (Gordon et al., 2020a).

Given that the primary outcome of these trials was AGAD symptoms, participants were not recruited based on depressive symptoms. At baseline, participants completed questionnaires including the 10-item GAD subscale of the Psychiatric Diagnostic Screening Questionnaire (PDSQ-GAD) (Zimmerman and Mattia, 2001), 16-item Penn State Worry Questionnaire (PSWQ) (Meyer et al., 1990), and several other measures of signs and symptoms of GAD (e.g., depressive symptoms) (Rush et al., 2003). Participants were classified as either AGAD (PDSQ-GAD ≥6 and PSWQ ≥45) or non-AGAD, and were diverted to parallel, identical RCTs evaluating the effect of the same RET program on AGAD symptoms in young adults either with or without AGAD. Participants were then randomized, stratified by sex and AGAD status, to RET or a wait-list control using www.randomizer.org. This study includes pooled data from both RCTs (i.e., AGAD and non-AGAD).

Inclusion criteria for both RCTs were: i) age 18–40y; ii) no medical contra-indication to participation in RET; and iii) no current pregnancy or lactation. Participants were not excluded for currently or previously engaging in RET or physical activity at or before baseline. To account for RET-specific familiarity and baseline physical activity levels, participants were asked, in reference to the time at baseline assessment, how long they had been involved in a formalized RET program; this was determined in weeks and used to quantify participant training age. Although not an exclusion criterion, no participants were currently involved in RET. RET and wait-list participants in both RCTs were advised to maintain their current levels of physical activity throughout the trial. Participants were not excluded for receiving other treatments for any mental health disorder. Ten participants were currently receiving treatment for depression (pharmacotherapy-only (*n* = 8), psychotherapy-only (*n* = 1), or both (*n* = 1)). No participants were receiving treatment for GAD. Four participants currently receiving treatment completed the trial (RET *n* = 2, wait-list *n* = 2); the other six withdrew (RET *n* = 2, wait-list *n* = 4).

This trial was originally powered to detect changes in anxiety and worry symptoms, which were the primary outcomes (Gordon et al., 2020a). Based on previous meta-analytic evidence of the small-to-moderate effect of RET on anxiety symptoms (Δ=0.31) (Gordon et al., 2017), and the moderate-to-large effect of RET on depressive symptoms (Δ=0.66) (Gordon et al., 2018), *a priori* power analyses were conducted using G*Power 3.1. These analyses indicated that for each parallel arm, sample sizes of 24 (12 per group) would provide >80% statistical power (two-tailed α=0.05, four repeated measures) to detect a small-to-moderate effect of RET on anxiety symptoms, and a slightly lower than anticipated (i.e., more conservative) small-to-moderate effect of RET on depressive symptoms.

### 2.3. RET intervention

RET was designed in accordance with WHO and ACSM guidelines (WHO, 2011; ACSM, 2019). The eight-week, twice-weekly intervention increased resistance progressively, such that the participant could complete two sets of between 8 and 12 repetitions before a deterioration in lifting form, or failure to complete a repetition. The investigator specified the resistance in accordance with guidelines rather than self-selection by participants. Load progressions were small and gradual. When participants completed two sets of 12 repetitions on an exercise, load was increased by 5% in the following session. When participants failed to complete two sets of at least 8 repetitions on an exercise, load was decreased by 5% in the following session. The eight exercises were barbell back squat, barbell bench press, hexagon bar deadlift, seated dumbbell shoulder lateral raise, barbell bent over rows, dumbbell lunges, seated dumbbell curls, and abdominal crunches. After baseline assessment, participants randomized to RET completed a three-week, twice-weekly familiarization process to ensure safety, correct lifting technique, and that the intervention was delivered at the correct resistance, starting at week one. Exercise sessions lasted approximately 25 min and were fully supervised on a one-to-one basis in a small, private, university-owned RET facility. All investigators were fully trained in delivering the exercise protocol consistently and identifying proper and improper lifting mechanics. Prior to RET sessions, participants completed primary and secondary outcome questionnaires in the RET facility. Further specifics of the RET intervention have been published previously (Gordon et al., 2020a).

### 2.4. Control condition

Participants randomized to the wait-list condition completed online questionnaires weekly, and were subsequently offered the RET intervention upon completion of their wait-list condition, but no data were collected.

### 2.5. Primary outcome

The primary outcome for this analysis was depressive symptom severity among the total sample. The 16-item self-report version of the Quick Inventory of Depressive Symptomatology (QIDS) (Rush et al., 2003) was used to assess depressive symptom severity at baseline, week one, four, and eight; symptoms were measured before the RET sessions. The QIDS has previously shown strong internal consistency (*a* = 0.86), and is a treatment-sensitive measure of depressive symptom severity (Trivedi et al., 2004). The internal consistency of the QIDS for the total sample was *a* = 0.77 (95%CI: 0.66 to 0.85). Correlations between repeated measures at baseline and week one were *r* = 0.67 (*p*<0.001) and *r* = 0.87 (*p*<0.001) for the RET and wait-list groups, respectively.

### 2.6. Covariates

Baseline physical activity was assessed using an online, self-report version of a seven-day Physical Activity Recall (Blair et al., 1985). Participants reported time engaged in sleep, and moderate, hard, and very hard activities during the prior week. Estimated energy expenditure was calculated as kilocalories per week. According to thresholds validated by Dishman and Steinhardt (1988) among young adult university samples, participants can be considered inactive (<245 kcal·kg^−1^·wk^−1^) to highly active (≥280 kcal·kg^−1^·wk^−1^).

### 2.7. Intervention fidelity and manipulation check

To calculate attendance percentage, the number of RET sessions attended was divided by 16 (two sessions per week for eight weeks) and multiplied by 100. To calculate compliance to the RET protocol, the number of sets in which at least eight repetitions were completed was divided by the total number of sets prescribed across the eight weeks (*n* = 256) and multiplied by 100.

To facilitate setting of the initial load, and to quantify anticipated changes in strength as a manipulation check, participants completed a five-repetition maximum (5RM) assessment for the barbell bench press, barbell back squat, and hexagon bar deadlift at baseline and postintervention. Total strength changes were measured as load changes on all three 5RM lifts combined. During the six familiarization sessions, participants completed two familiarizations with the 5RM process, and one maximal 5RM assessment.

### 2.8. Statistical analyses

Data analyses were performed using SPSS 26.0. Four percent of data were missing from the QIDS. Missing data were imputed for nine participants: sex and time-variant responses for each variable were entered as predictors into separate multiple linear regression models for condition, and predicted values were retained. Participants (*n* = 44) were excluded if they were missing primary outcome data for more than one time-point. These participants were predominately those who withdrew following randomization, but prior to completing any exercise or waitlist control condition protocol. Intention-to-treat analyses included all participants, and analyses of complete-cases only are reported as sensitivity analyses.

Independent samples *t*-tests examined baseline differences between conditions and sexes. The magnitudes of baseline differences were quantified using Cohen’s d effect sizes (Rosenthal et al., 1994). A two-group (RET/wait-list) x four time-point (baseline/week one/week four/week eight) repeated measures ANCOVA examined differences between RET and wait-list; age, sex, and baseline physical activity were covariates. A two-sex x two-group x four time-point repeated measures ANCOVA, adjusted for baseline physical activity, examined potential sex-related differences in the effects of RET compared to wait-list. The Huynh-Feldt adjustment was applied when the assumption of sphericity was violated. Significant interactions were decomposed using simple effects analyses. Standardized mean differences (SMD) quantified the magnitude of within-condition change. The magnitude of difference in outcome change between groups was quantified by Hedges’ *d* effect sizes by subtracting the mean change in the wait-list from the mean change in the RET condition and dividing this difference by the pooled standard deviation of baseline scores (Hedges and Olkin, 1985); effect sizes were adjusted for small sample size bias and calculated such that larger depressive symptom reductions among RET compared to wait-list resulted in positive effect sizes. Changes in strength were examined using paired-samples *t*-tests. Associations between changes in strength and changes in depressive symptoms were quantified using Pearson correlation coefficients of associations between change scores. Sub-analyses were performed for four subsamples: young adults with AGAD, without AGAD, with AMDD (QIDS ≥ 6), and without AMDD (QIDS ≤ 5).

## 3. Results

Tables 1 and 2 present baseline participant characteristics and changes in depressive symptoms across the trial, respectively, for the total sample and each subsample. There were no baseline differences between groups for any outcomes, supporting successful randomization; there were also no baseline differences between sexes for any outcomes (all *p*>0.14).

### 3.1. Intervention fidelity and manipulation check

The average attendance to the RET intervention was 83% (13 out of 16 sessions). The average compliance with RET was 80% (205 out of 256 sets). As previously reported, these attendance and compliance rates indicated that participants missed approximately three sessions over the course of the eight-week intervention, and were almost fully compliant when they attended. No adverse events arose from trial participation; however, one participant randomized to RET reported a headache during an exercise bout. The investigator supervising the bout immediately stopped the session, and the participant subsequently withdrew from the trial after consulting with a physician. The average 6–20 rating of perceived exertion was 14±1 (i.e., between somewhat hard and hard), and the average muscle soreness was 4 ± 2 (i.e., between mild and some soreness present) (Heath, 1998). As anticipated, participants in the RET intervention significantly increased their total strength (*t*(_20_)=−9.2, *p*<0.001, Cohen’s *d* = 2.01[95%CI: 1.25 *to* 2.75]), mean increase: 23.4%±13.2).

### 3.2. Depressive symptoms among the total sample

Table 2 presents descriptives, SMDs, and Hedges’ *d* [95%CI] effect sizes for changes in depressive symptoms from baseline. Results for males and females are reported in Table 1 in the Supplementary Materials.

At baseline, depressive symptom scores for the total sample were indicative of mild depression (QIDS: RET: 8.7; wait-list: 8.0; 6–10=mild depression). Among the total sample, no significant sex X group X time interaction was found for depressive symptoms (*F*(_2.3,114_)=0.942, *p* = 0.403). A significant group X time interaction was found (*F*_(2.3,113)_=10.18, *p*<0.001, *d* = 1.01[0.44 *to* 1.57]), such that RET significantly reduced depressive symptoms from baseline to week eight compared to the wait-list control (mean difference [*M_diff_*]=−5.82, *p*<0.001). When examining raw changes, depressive symptoms reduced among 39 participants (RET=24; wait-list=15) from baseline to week eight; symptoms increased (i.e., worsened) among eight participants (RET=2; wait-list=6), of which three transitioned from non-AMDD to AMDD (RET=2; wait-list=1), and did not change among eight wait-list participants. Findings were materially the same for intention-to-treat analyses (*d* = 0.88[0.45 *to* 1.31]) and analyses of complete cases only (*d* = 0.89[0.28 *to* 1.50]). Changes in strength were not significantly associated with changes in depressive symptoms (*r*_(21)_=−0.12, *p* ≥ 0.62).

### 3.3. Depressive symptoms and remission among AMDD, AGAD, and non-AGAD subsamples

Results for dichotomized subsamples (e.g., AMDD without AGAD) are reported in Table 2 in the supplementary materials. As would be expected, given the comorbidity of GAD and MDD, there is comorbidity in the current sample such that some participants were classified (and thus included in analyses) for both AGAD and AMDD.

Baseline depressive symptoms among the AMDD and AGAD samples were indicative of moderate depression (all QIDS>10). Among the AMDD sample, a significant group X time interaction was found for depressive symptoms (*F*(_2.5,80_)=10.72, *p*<0.001, *d* = 1.71[0.96 *to* 2.46]). RET significantly reduced depressive symptoms from baseline to week eight compared to the wait-list control ([*M_diff_*]=−7.73, *p*<0.001). RET reduced depressive symptoms among every member of the AMDD sample; 18 of the 19 (95%) RET participants remitted (QIDS<6) from AMDD by week eight. Among the wait-list (*n* = 18), 4 of 18 participants (22%) remitted from AMDD by week eight; symptoms remained the same among four, increased among four, and reduced without remission among six (33%).

Significant group X time interactions were also found for depressive symptoms among young adults with (*F*(_2.2,49_)=5.81, *p* ≤ 0.004, *d* = 1.39 [0.54 *to* 2.23]) and without (*F*_(2.7,62)_=5.46, *p* = 0.003, *d* = 1.24[0.43 *to* 2.05]) AGAD. RET significantly reduced depressive symptoms from baseline to week eight among those with ([*M_diff_*]=−7.81, *p<*0.001) and without ([*M_diff_*]=−4.13, *p*<0.001) AGAD compared to the wait-list control. Of the 27 participants with AGAD, 24 had comorbid AMDD.

Table 3 presents Hedges’ *d* effect sizes (95%CIs) for changes in depressive symptoms across successive measurement time-points.

## 4. Discussion

WHO and ACSM guidelines-based RET was an efficacious intervention for reducing depressive symptoms among young adults with limited RET experience. The efficacy of RET also appeared to be high among young adults with AGAD and AMDD, supporting RET as a promising treatment for mild or subclinical depression among young adults.

### 4.1. RET among the total sample

Attendance of 83% and compliance of 80%, and the absence of adverse events occurring from RET, supports that the RET intervention, which was representative of common, real-world RET practises, was feasible and tolerable. Further, a beneficial side-effect of RET was the large-magnitude increase in strength (*d* = 2.01); however, changes in strength were not associated with changes in depressive symptoms, and, consequently, RET that is programmed to improve strength may not be required for antidepressant benefits from RET among young adults with limited RET experience. Feasible, tolerable, and conservatively progressive RET that facilitates continuous engagement with RET activity may induce antidepressant benefits among young adults with limited RET experience.

Compared to a wait-list control group, RET significantly reduced depressive symptoms from baseline to week one (i.e., pre-post three-week familiarization protocol), and week four to week eight. Although reductions in depressive symptoms plateaued from week one to week four, the antidepressant effect of RET from baseline to week eight appeared larger than that of baseline to week four, and was materially the same among males (*d* = 1.11) and females (*d* = 0.89). Based on the threshold for clinical meaningfulness of at least half a standard deviation reduction from baseline scores (Norman et al., 2003), the large-magnitude reduction in depressive symptoms from baseline to week eight (*d* = 1.01[0.44 *to* 1.57]) is clinically meaningful. This antidepressant effect of RET is larger than previously reported findings for aerobic and resistance exercise (Δ=0.62) (Cooney et al., 2013), and for RET among older (≥55 years) adults (Δ=0.72) (Gordon et al., 2018), and comparable to that for mentally-ill adults (Δ=1.00) (Gordon et al., 2018). It is also larger than antidepressant effects of RET found in two previous experimental studies of young adults (*d* = 0.52, 0.84) (Herring et al., 2011; Vizza et al., 2016), but smaller than effects found in another trial of clinically depressed young adults (Δ=1.48) (Doyne et al., 1987). This is the third RET trial to report large-magnitude reductions in depressive symptoms among young adults, and the first to do so using ecologically-valid RET designed in accordance with WHO and ACSM guidelines.

The large-magnitude antidepressant effect found in this study supports RET as a promising treatment for mild or subclinical depression among young adults. This antidepressant effect is larger than those found for antidepressant medications, which have shown limited effectiveness for reducing mild-to-moderate depressive symptoms (Cohen’s *d* = 0.11) (Fournier et al., 2010), and are unlikely to be used for preventing the onset of clinical depression. The increased likelihood of young people who endorse depressed mood later developing clinical depression (odds ratio=2.52) (Wolitzky-Taylor et al., 2014) increases the potential importance of this finding. RET may be a particularly effective intervention for preventing the onset of clinical depression among individuals lower on the depressive symptom severity spectrum, while concurrently improving aspects of physical health and function.

### 4.2. RET among the AMDD and AGAD samples

The prevalent comorbidity of AMDD and AGAD in the current sample reflects the frequent comorbidity of GAD and MDD (Lieb et al., 2005). The current study is the first to investigate the antidepressant effects of ecologically valid, guidelines-based RET among young adults with clinically-meaningful symptoms of GAD, in whom depressive symptoms are frequently comorbid and may impact overall symptom severity, treatment compliance, and treatment response. RET induced large-magnitude, clinically-meaningful reductions in depressive symptoms from baseline to week eight among the AMDD and AGAD samples. Depressive symptoms among the AMDD and AGAD samples were similar to that of the total sample at week eight (QIDS: AMDD: 2.7; AGAD: 3.3; total: 2.8), and almost the entire AMDD sample (95%) remitted from AMDD. Consequently, RET reduced depressive symptoms among the AMDD and AGAD samples to the degree whereby those initially meeting criteria for AMDD no longer met those criteria by week eight.

The antidepressant effect of RET among the AMDD sample in this study is larger in magnitude than that of previous meta-analytic evidence for RET among participants with symptoms indicative of mild-to-moderate depression (Gordon et al., 2018). This antidepressant effect is also larger than those found in previous RCTs of young adults with major or minor depression (Doyne et al., 1987), and depressive symptoms indicative of mild-to-severe depression (Herring et al., 2011; Vizza et al., 2016). There are now four RCTs that have examined the effect of RET on depressive symptoms among young adults with either clinically-diagnosed depression, or symptoms indicative of at least mild depression. Three RCTs found large antidepressant effects (*d* = 0.84 to 1.71) using full-body RET among young adults with symptoms indicative of moderate-to-severe depression, while one found a moderate antidepressant effect (*d* = 0.52) using lower body RET among young adults with symptoms indicative of mild depression. Consequently, higher baseline depressive symptoms, using full-body RET, or both, potentially increase the effectiveness of RET for reducing depressive symptoms among young adults.

The antidepressant effect found among the AGAD sample is larger in magnitude than that of the only previous experimental trial to examine the effects of RET on depressive symptoms among young adults with clinically-meaningful anxiety (i.e., GAD) (Herring et al., 2011). Comorbidity is a fundamental characteristic of GAD, which is associated with an increased risk of depression (odds ratio=8.7) (Copeland et al., 2014). Such comorbidity among mental health disorders intensifies symptom severity, and makes treating both the primary and secondary disorder more difficult. The increased difficulty of treating comorbid mental health disorders adds significance to the reductions in both depressive and anxiety (*d* = 0.71) (Gordon et al., 2021) symptoms found among the AGAD sample in this RET trial. The large magnitude antidepressant effect of RET among those with AGAD suggests that RET may be a promising primary or adjunctive treatment for moderate depressive symptoms among young adults with clinically-meaningful anxiety, and little previous RET experience. The size of the large magnitude reductions in depressive symptoms among participants with AGAD (*d* = 1.39) could be due to their higher baseline depressive symptoms (QIDS: 11.4) (Gordon et al., 2018). There could also be a synergistic effect, whereby the simultaneous reductions in symptoms of GAD and depression augment each other, resulting in further symptom reductions. The overlapping aetiologies of GAD and MDD may explain these simultaneous antidepressant and anxiolytic effects. Greater psychosocial and psychobiological benefits of RET among those with a greater anxiety symptom burden may have also contributed to the larger-magnitude antidepressant effect among the AGAD sample.

### 4.3. Limitations and future research

Limitations of this study include the lack of an attention-control condition. Social interaction, attention, mastery experiences, and expectations for improvement, all of which cannot be controlled for with a non-active control group, may have contributed to the antidepressant effect found among the RET group. Still, the comparison to a non-active control gives a baseline indication of the efficacy of a RET intervention for reducing depressive symptoms. Future trials should use active attention-controls, such as minimal-intensity exercise, to examine the antidepressant effect of RET while controlling for non-exercise intervention components. Further, there were no post-intervention follow-ups in the current study; given the commonality of relapse following the cessation of treatment among patients with MDD (Ramana et al., 1995), long-term follow-ups are needed to examine whether depressive symptom reductions, and involvement in RET, are maintained. Future trials should also control for the use of contraceptive medications, due to their effects on reproductive hormonal balance. Inter-individual variation in depressive symptom responses to RET also remains unclear. As this was a pilot efficacy trial, though adequately powered, the sample size was small; future trials with larger sample sizes will allow for moderation and mediation analyses to examine such response variation. Using the QIDS as the diagnostic measure of depression status for the AMDD sample was also a potential limitation. The QIDS is primarily used to measure changes in symptoms across time, rather than diagnosing depression status. Future trials should use diagnostic questionnaires to diagnose depression status. The effect of RET on depressive symptoms among young adults with clinically-meaningful MDD with or without comorbid, clinically-meaningful GAD, remains unclear. Given the prevalence and additional burden of comorbid GAD and MDD, the associated increased treatment difficulty, and the potential synergistic effect, whereby simultaneous reductions in depressive and anxiety symptoms following RET augment each other, continued evaluation of RET as a treatment for comorbid GAD and MDD is warranted.

## 5. Conclusion

Eight weeks of ecologically-valid RET designed in accordance with WHO and ACSM guidelines resulted in clinically-meaningful, large-magnitude reductions in depressive symptoms among an otherwise healthy sample of young adults. Sub-analyses also revealed large anti-depressant effects among participants with AMDD and AGAD. There is a potential synergistic effect among those with AGAD, such that reductions in depressive and anxiety symptoms following RET augment each other. The large-magnitude increase in strength was a beneficial side-effect of RET, and was not associated with changes in depressive symptoms.

## Supplementary Material

Supplementary Materials

Supplementary material associated with this article can be found, in the online version, at doi:10.1016/j.psychres.2023.115322.

## Figures and Tables

**Table 1:** Total sample participant characteristics and baseline differences between groups.

	RET (*n* = 26)	WL (*n* = 29)			
Variables	Mean (SD)	Mean (SD)	*t*	*p*	Cohen’s d (95%CI)
**% Female**	65.4	62.1			
**Age (y)**	25.8 (5.7)	27.5 (5.7)	−1.1	0.271	−0.30 (−0.83 to 0.23)
**BMI**	24.6 (4.1)	24.5 (4.1)	0.1	0.893	0.02 (−0.51 to 0.55)
**Physical Activity (kcal·kg^−1^·wk^−1^)**	263.2 (30.2)	271.1 (43.9)	−0.8	0.442	−0.21 (−0.74 to 0.32)
**Depressive Symptoms (QIDS)**	8.7 (4.2)	8.0 (5.3)	0.6	0.578	0.15 (−0.38 to 0.68)

Physical activity was assessed via 7-day Physical Activity Recall. RET=Resistance Exercise Training; WL=Wait-list; SD=Standard Deviation; *y*=Years; BMI=Body Mass Index; QIDS=Quick Inventory of Depressive Symptomatology.

**Table 2:** Changes in depressive symptoms from baseline.

Sample	Group	*n*	Baseline	Week 1	SMD	Hedges’ *d* from baseline	Week 4	SMD	Hedges’ *d* from baseline	Week 8	SMD	Hedges’ *d* from baseline
**Total**	RET	26	8.7 (4.2)	5.2 (3.4)	0.92	0.59 (0.05 to 1.13)*	4.5 (3.0)	1.15	0.66 (0.13 to 1.22)*	2.8 (2.0)	1.79	1.01 (0.44 to 1.57)*
	WL	29	8.0 (5.3)	7.4 (5.5)	0.11		7.1 (6.0)	0.16		7.0 (5.5)	0.19	
**AMDD**	RET	19	10.6 (3.3)	6.2 (3.4)	1.31	0.90 (0.22 to 1.57)*	4.9 (2.8)	1.86	1.14 (0.45 to 1.84)*	2.7 (1.7)	3.01	1.71 (0.96 to 2.46)*
	WL	18	11.3 (3.9)	10.2 (5.0)	0.25		9.8 (6.1)	0.29		9.7 (5.2)	0.35	
**AGAD**	RET	12	11.4 (3.9)	7.1 (3.6)	1.15	0.79 (0.00 to 1.58)*	5.8 (2.7)	1.67	1.07 (0.26 to 1.88)*	3.3 (1.9)	2.64	1.39 (0.54 to 2.23)*
	WL	15	11.3 (5.0)	10.7 (5.2)	0.12		10.7 (6.3)	0.11		9.7 (6.1)	0.29	

AMDD=Analogue Major Depressive Disorder; AGAD=Analogue Generalized Anxiety Disorder; *n*=sample size; SMD=Standardized Mean Difference; RET=Resistance Exercise Training; WL=Wait-list;

* indicates a significant difference from baseline score in simple effects analysis.

**Table 3:** Effect of RET on depressive symptoms across successive time-points.

Sample	Hedges’ *d* Baseline to Week 1	Hedges’ *d* Week 1 to Week 4	Hedges’ *d* Week 4 to Week 8
**Total**	0.59 (0.05 to 1.13)*	0.09 (−0.44 to 0.61)	0.33 (−0.21 to 0.86)*
**AMDD**	0.90 (0.22 to 1.57)*	0.21 (−0.44 to 0.85)	0.44 (−0.22 to 1.09)*
**AGAD**	0.79 (0.00 to 1.58)*	0.28 (−0.49 to 1.04)	0.29 (−0.47 to 1.05)*

AMDD: Analogue Major Depressive Disorder; AGAD=Analogue Generalized Anxiety Disorder;.

* Indicates a significant difference from previous time-point in simple effects analysis.
