# Supplementary material for: Effects of resistance exercise training on depressive symptoms among young adults: A randomized controlled trial
Source: Psychiatry Res. Author manuscript; Available in PMC 2025 Jul 30. (PMC12309288; doi:10.1016/j.psychres.2023.115322)
Supplement: Supplementary Materials [file NIHMS2098348-supplement-Supplementary_Materials.docx]

**Supplementary materials**

**Table 1**

Changes in depressive symptoms from baseline among males and females in the total sample.

| **Sex** | Group | *n* | Baseline | Week 1 | SMD | Hedges’ *d* from baseline | Week 4 | SMD | Hedges’ *d* from baseline | Week 8 | SMD | Hedges’ *d* from baseline |
| --- | --- | --- | --- | --- | --- | --- | --- | --- | --- | --- | --- | --- |
| **Female** | RET | 17 | 8.5 (3.6) | 5.6 (3.7) | 0.80 | 0.45 (-0.21 to 1.11) | 4.5 (3.2) | 1.16 | 0.52 (-0.15 to 1.18) | 3.1 (2.2) | 1.84 | 0.89 (0.20 to 1.58)* |
|  | WL | 19 | 8.2 (5.5) | 7.5 (5.2) | 0.14 |  | 6.7 (5.9) | 0.26 |  | 7.1 (5.7) | 0.21 |  |
| **Male** | RET | 9 | 9.2 (5.5) | 4.6 (3.0) | 1.06 | 0.76 (-0.17 to 1.69) | 4.3 (2.5) | 1.15 | 0.89 (-0.05 to 1.83) | 2.2 (1.6) | 1.74 | 1.11 (0.14 to 2.07)* |
|  | WL | 10 | 7.6 (5.3) | 7.2 (6.2) | 0.07 |  | 7.7 (6.3) | -0.02 |  | 6.8 (5.5) | 0.15 |  |

RET=Resistance Exercise Training; WL=Wait-list; *n*=sample size; SMD=Standardized Mean Difference.

**p*<0.05

**Table 2**

Changes in depressive symptoms from baseline among dichotomized subsamples.

| **Sample** | *F* | *p* | Group | *n* | Baseline | Week 1 | SMD | Hedges’ *d* from baseline | Week 4 | SMD | Hedges’ *d* from baseline | Week 8 | SMD | Hedges’ *d* from baseline |
| --- | --- | --- | --- | --- | --- | --- | --- | --- | --- | --- | --- | --- | --- | --- |
| **AMDD without AGAD** | 6.396 | .002 | RET | 8 | 8.5 (1.9) | 4.6 (2.5) | 1.76 | 1.00 (-0.18 to 2.18)* | 3.6 (2.4) | 2.26 | 0.68 (-0.46 to 1.83)* | 2.4 (1.6) | 3.47 | 2.58 (1.09 to 4.08)* |
|  |  |  | WL | 5 | 7.6 (1.5) | 5.6 (3.2) | 0.80 |  | 4.0 (1.2) | 2.65 |  | 6.4 (2.6) | 0.57 |  |
| **AMDD with AGAD** | 7.719 | <.001 | RET | 11 | 12.1 (3.3) | 7.3 (3.7) | 1.37 | 1.11 (0.25 to 1.98)* | 5.9 (2.8) | 2.03 | 1.53 (0.62 to 2.44)* | 3.0 (1.8) | 3.42 | 2.06 (1.07 to 3.05)* |
|  |  |  | WL | 13 | 12.7 (3.6) | 11.9 (4.5) | 0.20 |  | 12.0 (5.7) | 0.15 |  | 11.0 (5.4) | 0.37 |  |
| **No AMDD or AGAD** | 1.611 | .207 | RET | 6 | 3.7 (1.4) | 2.3 (1.9) | 0.84 | 1.01 (-0.09 to 2.10) | 2.8 (3.4) | 0.35 | 0.61 (-0.45 to 1.66) | 2.3 (2.7) | 0.65 | 1.01 (-0.09 to 2.10) |
|  |  |  | WL | 9 | 2.7 (1.4) | 2.8 (2.1) | -0.06 |  | 2.7 (1.2) | 0.00 |  | 2.8 (1.7) | -0.06 |  |
| **AGAD without AMDD** | - | - | RET | 1 | - | - | - | - | - | - | - | - | - | - |
|  |  |  | WL | 2 | - | - | - |  | - | - |  | - | - |  |

AMDD=Analogue Major Depressive Disorder; AGAD=Analogue Generalized Anxiety Disorder; *F*=repeated measures ANCOVA *F* value for group x time interaction; *P*=significance of repeated measures ANCOVA group x time interaction; *n*=sample size; SMD=Standardized Mean Difference; RET=Resistance Exercise Training; WL=Wait-list; *indicates a significant difference from baseline score in simple effects analysis.
